# The association between parental internalizing disorders and child school performance

**DOI:** 10.1038/s41539-023-00182-x

**Published:** 2023-09-05

**Authors:** Magnus Nordmo, Thomas Kleppestø, Hans Fredrik Sunde, Martin Flatø, Perline Demange, Fartein Ask Torvik

**Affiliations:** 1 Department of Educational Science, University of South-Eastern Norway, Notodden, Norway; 2 Centre for Fertility and Health, Norwegian Institute of Public Health, Oslo, Norway; 3 Department of Biological Psychology, Vrije Universiteit Amsterdam, Amsterdam, The Netherlands; 4 Promenta Research Center, Department of Psychology, University of Oslo, Oslo, Norway

**Keywords:** Education, Human behaviour

## Abstract

Parents play a crucial role in children’s lives. Despite high prevalences of anxiety and depression, we do not know how these disorders among parents associate with child school performance in Norway. We use regression models to estimate associations between parental mental disorders and child school performance, while adjusting for some social and genetic confounders. Parental anxiety and depression were assessed from administrative registers of government funded health service consultations for all individuals in Norway with children born between 1992 and 2002. School performance was assessed as standardized grade point average at the end of compulsory education when children are 16 years old. Associations were also considered in samples of adoptees and among differentially affected siblings. We find that 18.8% of children have a parent with an anxiety or depression diagnosis from primary care during the last three years of compulsory education (yearly prevalence: 11.5%). There is a negative association between these parental mental disorders and child school outcomes (*z* = 0.43). This association was weakened, but statistically significant among differentially exposed siblings (*z* = 0.04), while disappearing in adoptee children. Many children experience that their parents have anxiety or depression and receive a diagnosis from primary care. On average, these children have lower school performance. The association is attenuated when comparing differentially exposed siblings and disappears in adoptee children. These results have a poor fit with the hypothesis that parental internalizing is an influential causal factor in determining children’s educational success.

## Introduction

Parenthood is a formidable task. Few of life’s challenges compare in terms of sustained effort. Unfortunately, mental health has shown to be negatively affected by the transition to parenthood and many parents suffer from mental disorders^1^. The impact of parental mental disorders on child development has been studied extensively^2–4^, with a subset of studies focusing on children’s school outcomes^5–8^. These studies are compelling as children’s school performance has been linked to health, economic prosperity, and well-being^9–11^. However, studies on internalizing disorders, which are common in the population, have limitations related to representativeness and confounding. Given that parents provide the social and emotional context in which the child develops, there is a potential for parental mental disorders to negatively influence child school performance. Supporting this notion are studies showing that parental involvement is associated with better learning strategies^12^ and generally benefit children’s school outcomes^13^. If parent’s ability to assist is compromised due to poor mental health, then this can have a negative impact on the child’s educational success. Parental mental health can thereby play a role in driving social inequality if children’s educational outcomes are negatively affected.

Studies on parental mental health and child school performance consist of two main areas of research. One area of research focuses on severe mental disorders such as bipolar disorder and schizophrenia. These studies indicate that severe parental mental disorders are negatively associated with child school performance and associated with higher probability of dropping out of compulsory education^14–16^. These observed associations are pronounced and robust to adjustments for socioeconomic status. Another area of research investigates maternal depression and its various effects on child outcomes. Augustine & Crosnoe^5^ found that self-reported maternal depression predicted lower scores on standardized tests during the first years of primary school but only for children with mothers without higher education. Claessens et al.^17^ showed that self-reported persistent maternal depression is associated with worse outcomes on standardized tests of language, literacy and mathematics in the first years of formal schooling. Adjusting for a range of confounders such as family income and maternal education, they conclude that the conditional association between maternal depression and test scores was one tenth of a standard deviation.

Others have investigated timing effects of parental mental disorders on child school performance. Both Shen et al.^8^ and Brophy et al.^7^ found modest negative associations of parental depression, as diagnosed by specialist and primary care, respectively. Brophy et al.^7^ assessed educational attainment at 6/7-, 10/11-, and 13/14-year-olds while Shen et al.^8^ investigated educational success at age 16. The associations were lessened but remained significant after adjusting for socioeconomic factors. The authors measured parental depression at several time points during the child’s development but did not find a critical period. They concluded that the link between parent mental health and child education had shorter and longer pathways of influence. The authors note that parental depression before the birth of the child is equally associated with children failing school. This observation is consistent with developmental theories that encompasses both biological and social factors.

Previous literature has adjusted the association between parental disorders and child school performance for observable socio-economic characteristics, to account for that parental income or education may confound this relationship^5,7,8,17^. An alternative approach is the sibling comparison method. A sibling who experiences parental mental illness before receiving their final grades can be compared to an older sibling who had already finished school, as a counterfactual comparison. The benefit of this approach is that it controls for confounding factors that are either constant across time or randomly received by either sibling. For this reason, sibling comparison studies can reduce confounding bias, also when confounding is caused by unobserved characteristics^18^. Another approach to untangle genetic confounding is to restrict the sample to adoptee children. As far as we are aware, no studies have previously investigated this relationship using these two methods.

In summary, the studies that focus on parental depression show small associations that are appear robust to confounding on observed social factors. These associations have been documented with both clinical diagnoses and self-reported measures. Whether parental mental disorders have a causal effect on child school performance remains unknown due to possible social and genetic confounding. The aim of this study is to investigate the association between parental mental disorders and child school performance using population-wide diagnostic data with a genetically informed design. The goal is trifold: First, identify the proportion of children who experience a parent with a diagnosis related to internalizing disorders (anxiety and depression). Second, to investigate the strength of the relationship before and after adjusting for known observed confounders. Third, to investigate the strength of the relationship after adjusting for unobserved time-constant confounders, including genetic influences, by using data from siblings and adoptees.

## Results

### Prevalences

We found that 18.8% of children had experienced a parent with an internalizing disorder diagnosis in the last three years of primary education. In an average year between 2006–2018, 11.5% of children had a mother (7.9%) or a father (4.3%) with an internalizing disorder. The average annual prevalence for any parental mental disorder, in either the mother, the father, or both, was 14.4%. Table 1 shows diagnosis prevalence for each disorder. Most parents with an internalizing diagnosis had recurrent diagnoses for several years. Only 10.4% of parents with an internalizing disorder had a single year with a diagnosis while the remaining parents had multiple years with a registered diagnosis.

**Table 1:** Average Yearly Prevalence from 2006–2018.

	Either		Mother		Father	
Disorder	*N*	Percent^a^	*N*	Percent^a^	*N*	Percent^a^
Any psychiatric	96,031.6	14.4	65,129.9	9.8	37,951.1	5.7
Internalizing	76,626.1	11.5	52,201.0	7.9	28,825.1	4.3
Depressive disorder	60,036.3	9.0	40,550.5	6.1	22,321.8	3.3
Anxiety disorder	19,592.4	3.0	13,101.6	2.0	6,830.2	1.0
Phobia/Compulsive disorder	4394.1	0.7	2656.4	0.4	1759.2	0.3
Drug abuse	3844.0	0.6	1433.7	0.2	2654.5	0.4
Chronic alcohol abuse	5116.5	0.8	1453.1	0.2	3701.2	0.6
Medication abuse	1758.4	0.3	984.4	0.1	800.4	0.1

^a^Denominator (*N* Children): 667,955.

In the adoptee sample, 15.7% of children experienced a parent with an internalizing disorder the last three years of primary education, which is somewhat lower than in the total sample. A chi-square test of independence showed statistically significant different rates of parental internalizing disorder between the population and sibling sample, *x*^2^ (1, *N* = 741704) = 10179, *p* < 0.01, and between the population and adoptee sample, *x*^2^ (1, *N* = 673144) = 69.5, *p* < 0.01. See supplemental material for diagnosis frequencies in the adoptee sample.

### Association between parental internalizing and child GPA

**Fig. 1:**
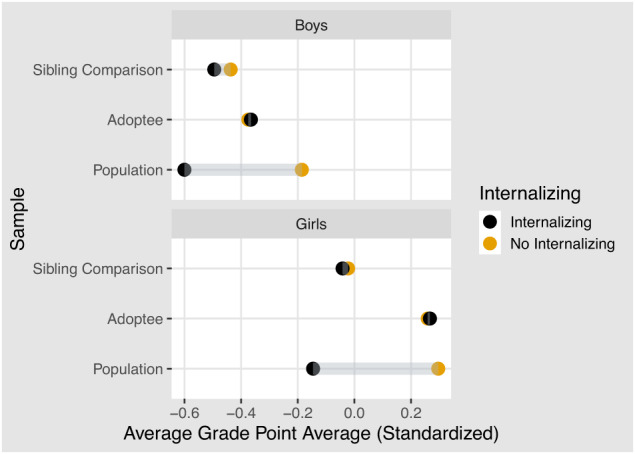
Average GPA for children with and without parental internalizing disorders. The two panels show averages for boys and girls. The vertical axis separates the three samples: the sibling comparison, the adoptees, and the population. The black dot represents children who experience at least one parent diagnosed with an internalizing disorder. The yellow dot represents the average value for children who do not experience parental internalizing.

**Fig. 2:**
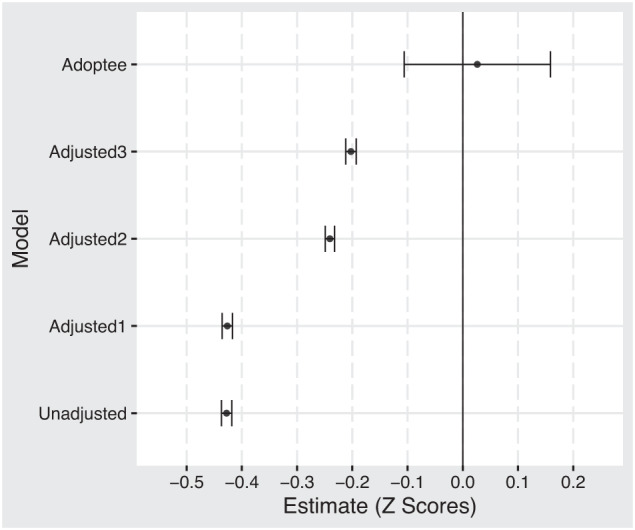
Model estimates from regression analysis. Model 1 is adjusted for birth order and birth year. Model 2 builds on model 1 and includes covariates for socioeconomic status. Model 3 builds on model 2 and includes covariates for all other psychiatric diagnoses and drug/alcohol disorders. The adoptee model is adjusted for socioeconomic status.Confidence interval set to 95%.

**Fig. 3:**
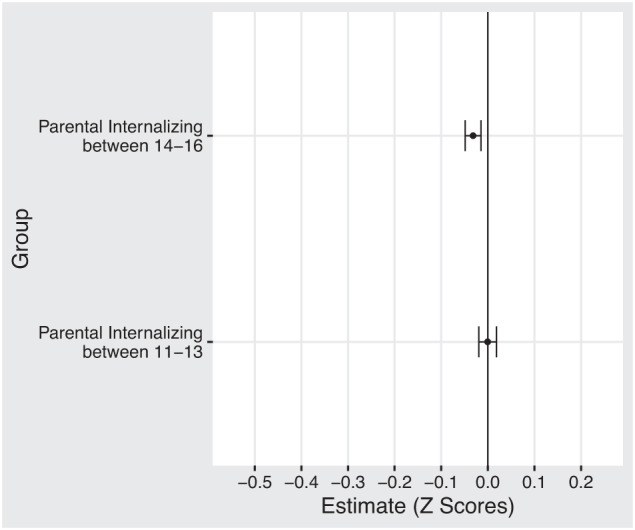
Sibling comparison estimates from fixed effects model. Sibling exposed between 17–19 serves as the comparison group. This group is compared with siblings who experience parental internalizing between 11–13 and 14–16. Both models are adjusted for birth order and birth year. Confidence interval set to 95%.

On average, children who experience a parental internalizing disorder receive a lower GPA. The difference is pronounced in the population sample but mostly disappears in the adoptee and sibling comparison samples. Figure 1 compares average GPA scores across the different samples, and Fig. 2 displays the results from the regression models. We found modest but statistically significant associations between child GPA and parental internalizing disorder in both population and sibling models. The largest association was found in the unadjusted model −0.43 [95 % CI: −0.44, −0.42]. Adjusting for child birth order and birth year (adjusted model 1) did not influence the association −0.43 [95% CI: −0.44, −0.42]. Adding parental socioeconomic status (adjusted model 2) reduced the association considerably (*β* = −0.24; 95% CI: −0.25, −0.23). We found a further reduction when adjusting for all other parental mental disorder and drug and alcohol use (adjusted model 3: *β* = −0.20; 95% CI: −0.21, −0.19). The results from the adoptee subsample did not show a statistically significant association (*β* = 0.03; 95% CI: −0.11, 0.16). A summary of all regression models is presented in the supplemental material and in Fig. 2. The results from the sibling comparison model suggests no significant difference between siblings exposed at age 11–13 versus age 17–19 (early exposure). However, we do find a significant difference between siblings exposed at age 14–16 versus age 17–19 (*β* = −0.03; 95% CI: −0.05, −0.01). See Fig. 3. Adjusted estimates for all ICPC-2 mental disorders are included in the supplemental material.

## Discussion

We corroborate earlier results that parental internalization is common and strongly associated with school performance. However, the association is much weaker when comparing siblings who were differentially exposed to mental disorders in parents and disappears when considering adopted children who are not genetically linked to their parents. Overall, our extensive adjustments are still consistent with a causal effect of parental internalizing disorders on child school performance yet puts into question whether such effects are large enough to be an important factor in the intergenerational transfer of social disadvantage.

Previous literature has speculated that associations between mental illness in parents and school performance are affected by genetic confounding^7,8,17^. We find associations comparable to the previous literature which do not control for unobserved social and genetic confounding, while the sibling comparison models have a much weaker association between parental internalizing disorders and child school performance. No statistically significant association was found in the adoptee analysis. The potential genetic confounding is highlighted by findings which reveal a genetic component in both mental health and school performance^19,20^. The nature of this shared genetic risk needs to be explored in future research.

Our sibling model reveals a small effect when the child experiences the parental internalizing disorders while in lower secondary school (aged 14–16). In contrast, we do not find a significant association if the child experiences parental internalizing disorders before this age (11–13). Considering that siblings who had already finished lower secondary when the parent was diagnosed may also have partly suffered from parental mental health problems, this suggests a likely lower bound on a possible causal effect (*z* = 0.04) which is about 1/7 of the total association (*z* = 0.28). This is small compared to other factors associated with school performance, for instance, the gender difference is 15 times larger (*z* = 0.476). The pervasiveness of parental internalizing disorders means that any negative effects on child outcomes is far-reaching, even with small effect sizes. One way forward is to employ a randomized controlled trial of parental mental health services and assess offspring outcomes, including educational performance. Readers should note that our estimates indicate that parental internalizing disorders are not a main driver of social inequalities.

Epidemiological studies^21,22^ report higher yearly prevalence of both depression (7.2%) and anxiety (6.5%) compared to our findings. This discrepancy is likely due to the characteristics of primary care data, compared to epidemiological surveys. Many parents with internalizing disorders do not seek treatment or might not receive the diagnosis in primary care. We find that mothers experience nearly double rates of internalizing disorders compared to fathers which also match with findings from epidemiological research^21^. Our findings should be evaluated in light of the heterogeneity found in both anxiety and depression diagnoses^23,24^. Different diagnostic practices and study populations will impact the association between parental anxiety/depression and child educational outcomes. Future research would benefit from a measure of severity of internalizing disorders.

Our study has three main contributions: First, we show that internalizing disorders are highly prevalent among parents. Second, internalizing disorders in parents mark a risk of low school performance. Third, the association is weakened in analyses of differentially exposed siblings, while disappearing in genetically unrelated adoptive children. If there is an effect from parental internalizing disorders to offspring educational success, then this association appears too small to be considered a main driver of societal inequalities.

This study has several notable strengths. Unlike some previous investigations, our design allows us to probe a range of potential sources of confounding including parental income and education levels, which has been shown to be associated with both child educational success and parental mental health^25,26^. The use of both adoptee and sibling analyses brings nuance to the question of genetic confounding which was lacking in the current literature. The sibling comparison implicitly controls for social factors that are identical across siblings^27^. We furthermore measure the association with parental internalizing disorders using administrative registries, circumventing selection bias^28^. The inclusion of both anxiety and depression allows us to capture a broad concept of internalizing disorders with diseases that are highly co-morbid.

Nevertheless, some limitations should be noted. First, not all individuals with internalizing disorders are recoded with primary care diagnoses. Nevertheless, primary care diagnostic data encompasses more mental disorder compared to data collected from specialist services, which has typically been used in register studies. In particular, symptoms of drug and alcohol abuse are underreported^29^. Second, adoptee status is not a random variable in the population. There are qualifications criteria that exclude people who cannot provide adequate care, in addition to self-selection effects. One example is the low rates of parental drug- and alcohol abuse diagnoses in parents that adopt, compared with the non-adoptee parents. Although there are many cases of parental internalizing disorders in the adoptee sample, these disorders might be less severe or shorter lasting compared to the population. The adoptee parents could also have access to resources which might lessen the impact of parental internalizing disorders on child school success. Only foreign-born adoptee children were included in the adoption study. The goal of this strategy was to remove the genetic pathway between parental internalizing and offspring educational success. Intra-country adoption in Norway is mainly adoption of relatives or stepchild. Data from such adoptees is therefore not informative for this aim. Third, in most cases, the differentially exposed siblings may all to some extent experience the parent suffering from mental illness. In many cases, the mental illness would have started long before the diagnosis. The effect of parental mental disorders might have long and diffuse causal pathways that affect child school performance. Both anxiety and depression are persistent phenomena where relapse and cyclical patterns are the norm^30,31^. Highly frequent and persistent parental internalizing disorders are less likely to differentially effect different siblings but is associated with school absence and worse school achievement^17^. Given these limitations, we interpret the sibling model as a lower bound of an acute effect, in contrast to the many possible long-term effects. The adoptee and sibling analyses suffer from methodological issues. These methods still provide results pointing in the same direction. Forth, we could not assess whether the internalizing disorder has remitted. Our analyses assumes that a year without a diagnosis indicates remission/no disease which could falsely categorize parents who experience internalizing disorder but do not seek medical consultation. Finally, we cannot rule-out reverse causality, where child school performance causes the parental internalizing disorder.

## Methods

### Sample

**Fig. 4:**
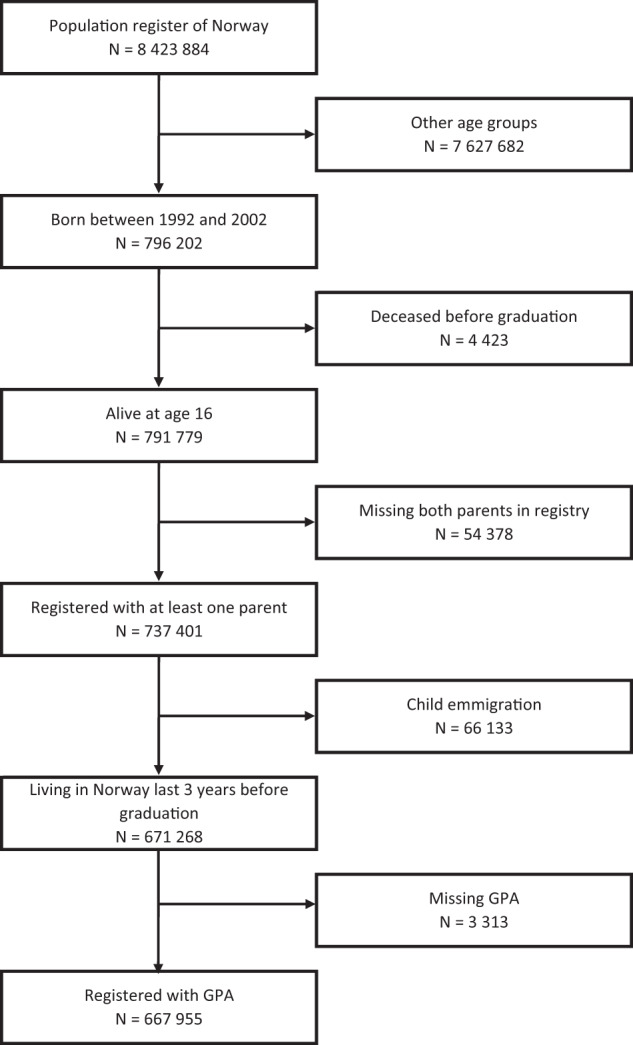
Sample determination flowchart. The flowchart details how we utilize the population register of Norway and end up with our study sample.

The population sample is based on the population register of Norway and consists of all children born between 1992 and 2002, participating in compulsory education, registered with GPA between age 15 and 17, and with at least one known parent. A flowchart of sample selection is shown in Fig. 4. We selected this timespan because it allowed us to combine data on child education and parental health. As this is a registry study of the population, we could not collect informed consent from the study participants.

#### Previous diagnoses covariate sample

In our analysis, we run a model (adjusted model 3) which controls for the confounding factors of all other parental mental disorder diagnoses, as well as parental drug and alcohol abuse while the child was between 11 and 13 years old. This results in a slightly smaller sample (*n* = 607,795) as the available primary care data starts in 2006. The aim of this analysis is to isolate the effect of internalizing disorders from previous comorbid mental illness diagnoses, as well as drug and alcohol abuse. We chose the age-period between 11–13 as we did not want to remove the possible mediating pathway from parental internalizing disorders, through other concurrent or later conditions, into offspring educational success.

#### Sibling sample

The fixed effect sibling model relies on the subset of siblings with unique exposures to parental mental disorder during lower secondary school. We identified family clusters using birth records where each child receives a family-unique ID based on having the same mother and father. We analyzed a subgroup of siblings (*n* = 73,740) where one sibling was exposed to parental mental disorders during primary or lower secondary school, with at least one comparison sibling with exposure to parental internalizing disorder between age 17–19 years (i.e., after graduation). The comparison sibling group was not exposed to parental internalizing disorders between 11–16 years old.

#### Adoptee sample

We also estimated associations among foreign adopted children. We inferred adoptee status by identifying children who were not themselves born in Norway, but whose parents were living in Norway at the time the child was born. We only included children from non-western countries that are common origin countries for adoptees (*N* = 5189): Colombia, China, South-Korea, India, Ethiopia, Philippines, Brazil, Thailand, Russia, Romania, Chile, Bolivia, Ecuador, Vietnam, Singapore, Peru, Guatemala, and Sri-Lanka. The birth countries of the adoptee sample mirrored adoption rates obtained from official adoptee registers.

### Measures

#### School performance

Norwegian students are evaluated after ten years of compulsory education, usually the year they turn 16. In the period 2009–2015, only 3.5% of the students did not receive a GPA due to few evaluations^32^. Among individuals with a registered GPA, 98% (*n* = 654,515) had the GPA registered the year they turned 16 (year turning 15: 0.3%; *n* = 1 891, year turning 17: 1.3%; *n* = 8949). A very small proportion of students had graduation earlier than 15 (*n* = 16) or later than 17 (*n* = 2584). These grades have marks from 1 to 6, where 6 is best. The GPA is calculated as the average of all final-year teacher evaluated grades and externally graded exams. The GPA is used for ranking students applying for admission to upper secondary education. Students therefore have an incentive to perform well. We standardized the GPA score (mean = 0, SD = 1) within each birth year cohort to adjust for grade inflation. It is not possible to fail the compulsory education in Norway and all students progress through mandatory education at the same pace. Even the lowest grades go into the GPA score, also those that would not be considered passing at a higher level of education. This means that nearly all students have a valid GPA. A school subject is marked as “not evaluated” if the teacher cannot evaluate the student due to absence, or in special circumstances related to learning difficulties or non-native speakers. If more than half of the subjects are “not evaluated”, the student will not receive a GPA.

#### Diagnoses of mental disorders

All individuals who legally reside in Norway have access to a general practitioner. Most patients are registered with the general practitioner, because making use of specialist health care requires a referral from a general practitioner. The service is free of charge for children and adolescents under 16 and heavily subsidized for adults. General practitioners send billing information to The Norwegian Health Economics Administration (HELFO) along with a diagnosis or reason for the visit to receive reimbursements. Due to these economic incentives, it is unlikely that visits to general practitioners go unreported. Diagnostic information is coded according to the International Classification of Primary Care, 2nd edition (ICPC-2)^33^. To capture parental internalizing disorders, we collapse the diagnostic codes P74 Anxiety Disorder, P76 Depressive Disorder, and P79 Phobia/Obsessive Compulsive Disorder. Drug and alcohol abuse symptoms were captured with the ICPC-2 symptom codes P15 Chronic Alcohol Abuse, P18 Medication Abuse, and P19 Drug Abuse. Similarly, when adjusting for all mental disorders we include all ICPC-2 diagnoses from P70 (Dementia) to P99 (Psychiatric Disorder Unknown).

We utilized diagnostic data from 2006 to 2018. For the exposure of interest in the main analysis, a diagnosis was coded as 1 if there was a parental diagnosis within three years of the child’s graduation (14–16 years old). A 0 was coded if no diagnosis occurred within this timeframe. For our ICPC-2 covariates, we utilized mental disorder diagnoses and alcohol/drug use symptoms when the child was between 11 and 13 years old, i.e. prior to the exposure of interest^34^.

#### Income and education

Income data were obtained from tax records and included income from wages, self-employment, capital income, pensions, and government assistance such as disability benefits collected the year the child turned 12. Parental education level was collected from the National Education Database recorded the year the child turns 12. We coded parental education as a categorical variable of highest achieved educational level following the International Standard Classification of Education; Lower Secondary Education (1), Upper/Post-Secondary Education (2), Tertiary Undergraduate (3), and Tertiary graduate and above (4). In total, 4.6% parental income and 4.7% education data were missing. We imputed the gender specific median income (male: 386 274, female: 261 488) and education (male: 2, female: 2) to include all cases in our analyses.

### Statistical analyses

First, we estimated the unadjusted associations between being exposed to a parental internalizing disorder (either parent) during the three years leading up to graduation and GPA (unadjusted model). We then adjusted the estimate by adding child birth order and birth year as factors (adjusted model 1). Next, we added parental income and education (adjusted model 2). We further added all other parental psychiatric diagnoses, as well as drug and alcohol use as independent variables (adjusted model 3).

We supplemented these analyses with two approaches. We analyzed the adoptee sample using parental internalizing disorders, parental income and education, birth order, and birth year as predictors. Next, we estimated a fixed-effect sibling comparison model. We separated siblings who experienced parental internalizing disorders into three groups. Those experiencing parental internalizing disorders between 11–13, 14–16, and 17–19 years old. The latter was utilized as a comparison group as GPA is set at 16. We also included birth order and birth year as covariates. We ran the fixed effect model without other predictors because these are typically identical between siblings. We also ran distinct models separating mother and father internalizing disorders, as well as parental depression and anxiety. These are presented in the supplemental material.

### Reporting summary

Further information on research design is available in the Nature Research Reporting Summary linked to this article.

### Supplementary information

Supplemental Material

Reporting Summary
