# Author Correction: The association between parental internalizing disorders and child school performance

**DOI:** 10.1038/s41539-023-00195-6

**Published:** 2023-09-29

**Authors:** Magnus Nordmo, Thomas Kleppestø, Hans Fredrik Sunde, Martin Flatø, Perline Demange, Fartein Ask Torvik

**Affiliations:** 1 Department of Educational Science, University of South-Eastern Norway, Notodden, Norway; 2 Centre for Fertility and Health, Norwegian Institute of Public Health, Oslo, Norway; 3 Department of Biological Psychology, Vrije Universiteit Amsterdam, Amsterdam, The Netherlands; 4 Promenta Research Center, Department of Psychology, University of Oslo, Oslo, Norway

**Keywords:** Education, Human behaviour

Correction to: *npj Science of Learning* 10.1038/s41539-023-00182-x, published online 05 September 2023

The Acknowledgements section was missing from this article and should have read ‘This work was supported by the Research Council of Norway (grants number 300668). This work was partly supported by the Research Council of Norway through its Centers of Excellence funding scheme (grant number 262700)’.

