# Supplementary material for: The association between parental internalizing disorders and child school performance
Source: NPJ Sci Learn. 2023 Sep 5;8:34. doi: 10.1038/s41539-023-00182-x (PMC10480151; doi:10.1038/s41539-023-00182-x)
Supplement: Supplementary file 1 — Supplemental Material [file 41539_2023_182_MOESM1_ESM.pdf]

Supplementary Information

Sensitivity Analyses

In addition to the analyses presented in the main manuscript, we performed several sensitivity analyses in order to investigate subgroups. First, we separated internalizing into depression and anxiety. We utilized the same procedure as presented in the main manuscript but included depression and anxiety as predictors in separate models. These estimates are shown below in Supplemental Figure 1. See the end of this document for a table of model summaries.

Supplemental Figure 1

Model Estimates Depression and Anxiety

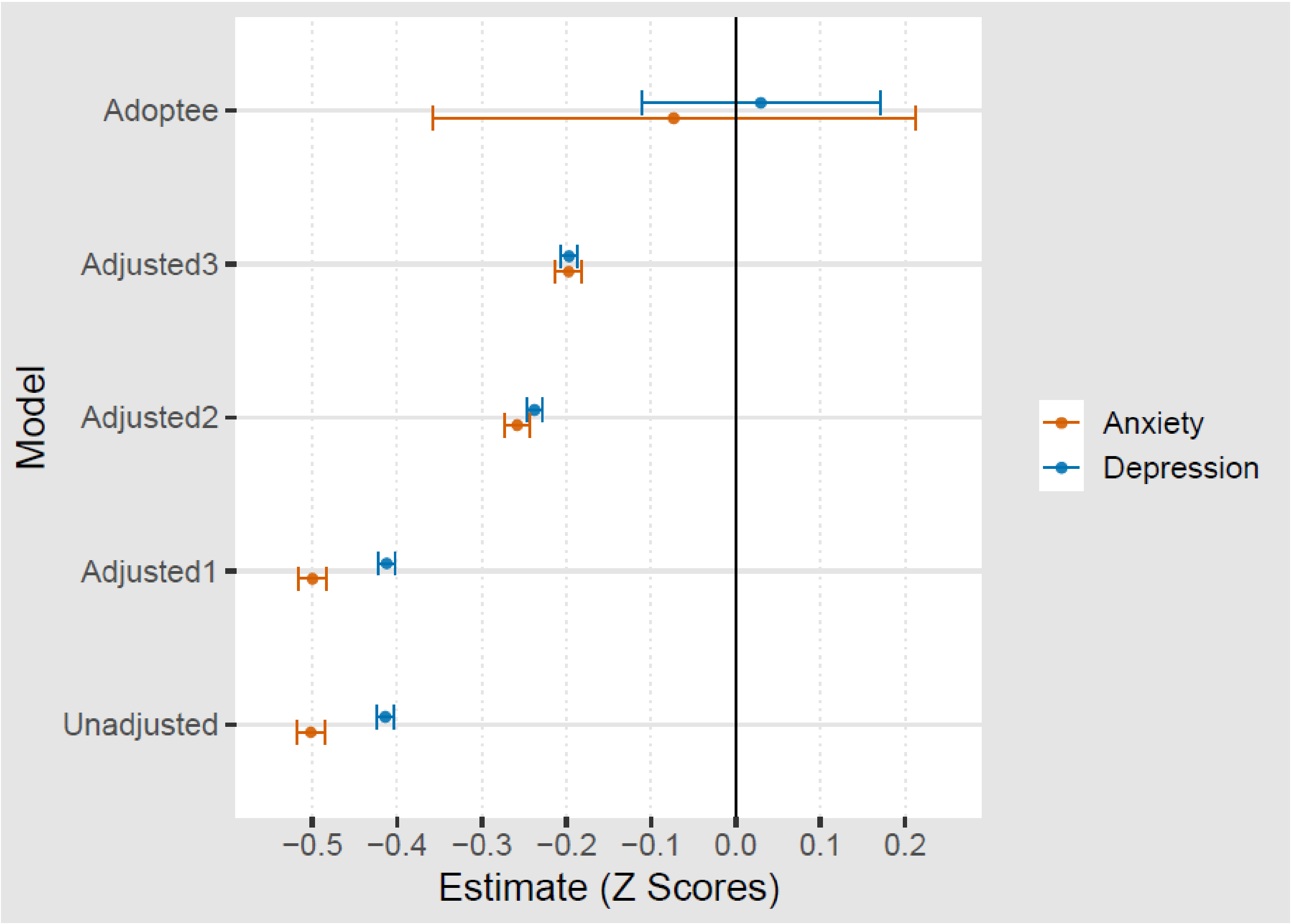

Adjusted1: Adjusted for birth order and birth year
Adjusted2: Adjusted 1 and covariats for socioeconomic status
Adjusted3: Adjusted 2 and covariats for all other psychiatric diagnoses and drug/alcohol disorders
Adoptee: Adjusted for socioeconomic status
95 % Confidence Intervals

Second, we repeated the main analysis but utilized only mother and father internalizing in separate models. These estimates are shown below in Supplemental Figure 2.

Supplemental Figure 2

Model Estimates Father and Mother Internalizing

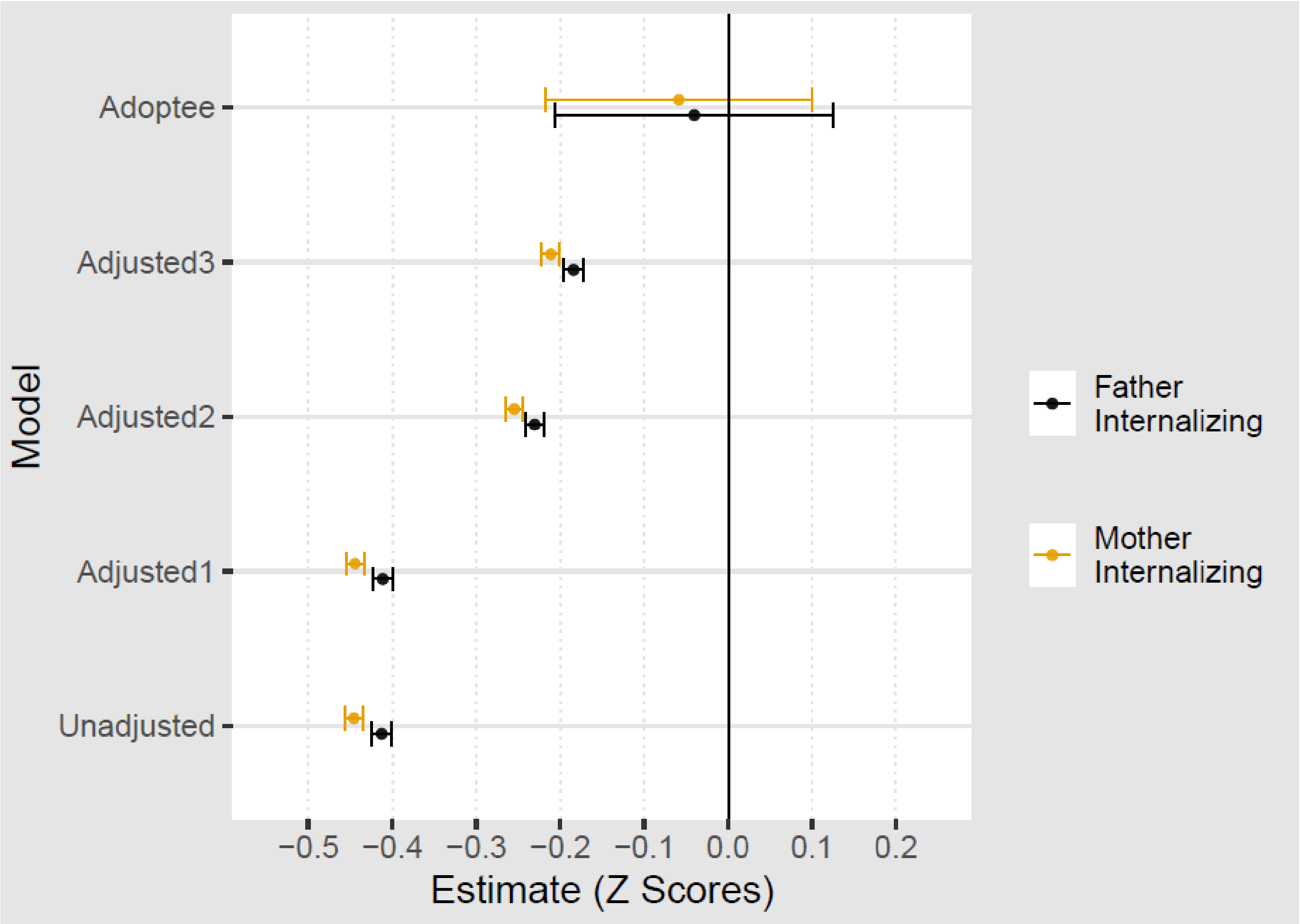

Adjusted1: Adjusted for birth order and birth year
Adjusted2: Adjusted 1 and covariats for socioeconomic status
Adjusted3: Adjusted 2 and covariats for all other psychiatric diagnoses and drug/alcohol disorders
Adoptee: Adjusted for socioeconomic status
95 % Confidence Intervals

Adoptee Sample

As shown in Supplemental Table 1, the adoptee sample show less parental internalizing disorders compared with the population percentages. Also, drug and alcohol abuse diagnoses are very low which is expected given the screening procedures for these disorders in adoption routines.

Supplemental Table 1

Average Yearly Prevalence in Adoptee Sample

	Either		Mother		Father	
Disorder	N	Percent[1]	N	Percent[1]	N	Percent[1]
Any Psychiatric	553.2	10.7	389.7	7.5	190.1	3.7
Internalizing	478.2	9.2	338.2	6.5	160.1	3.1
Depressive Disorder	380.6	7.3	269.2	5.2	125.2	2.4
Anxiety Disorder	107.2	2.1	73.6	1.4	34.4	0.7
Phobia/Compulsive Disorder	26.4	0.5	17.4	0.3	9.0	0.2
Chronic Alcohol Abuse	26.9	0.5	< 10	< 0.17 %	21.1	0.4
Medication Abuse	< 10	< 0.17 %	< 10	< 0.17 %	< 10	< 0.17 %
Drug Abuse	< 10	< 0.17 %	< 10	< 0.17 %	< 10	< 0.17 %

[1]Denominator (N Children): 5 189

Supplemental Table 2

Internalizing Regression Coefficients

	GPA			GPA			GPA			GPA			GPA		
Predictors	*Estimates*	*CI*	*p*	*Estimates*	*CI*	*p*	*Estimates*	*CI*	*p*	*Estimates*	*CI*	*p*	*Estimates*	*CI*	*p*
(Intercept)	0.05	0.05 – 0.05	**<0.001**	0.03	0.02 – 0.04	**<0.001**	-1.19	-1.20 – -1.18	**<0.001**	-1.89	-1.94 – -1.84	**<0.001**	-0.40	-0.52 – -0.27	**<0.001**
Internalizing	-0.43	-0.44 – -0.42	**<0.001**	-0.43	-0.44 – -0.42	**<0.001**	-0.24	-0.25 – -0.23	**<0.001**	-0.20	-0.21 – -0.19	**<0.001**	0.03	-0.11 – 0.16	0.697
Birth order [2]				0.02	0.01 – 0.02	**<0.001**	-0.02	-0.03 – -0.02	**<0.001**	-0.02	-0.03 – -0.02	**<0.001**	-0.06	-0.13 – -0.00	**0.049**
Birth order [3]				-0.00	-0.01 – 0.01	0.494	-0.04	-0.05 – -0.03	**<0.001**	-0.05	-0.06 – -0.04	**<0.001**	-0.40	-0.61 – -0.20	**<0.001**
Birth order [4]				-0.19	-0.22 – -0.16	**<0.001**	-0.11	-0.14 – -0.08	**<0.001**	-0.11	-0.14 – -0.08	**<0.001**	-0.46	-1.40 – 0.47	0.329
Birth order [5]				-0.31	-0.39 – -0.24	**<0.001**	-0.10	-0.17 – -0.04	**0.002**	-0.10	-0.17 – -0.04	**0.002**			
Birth order [6]				-0.28	-0.43 – -0.13	**<0.001**	-0.07	-0.21 – 0.06	0.300	-0.07	-0.21 – 0.06	0.291			
Birth order [7]				-0.15	-0.49 – 0.19	0.398	0.07	-0.24 – 0.37	0.680	0.05	-0.25 – 0.36	0.728			
Birth order [8]				-0.51	-1.19 – 0.17	0.145	-0.35	-0.97 – 0.27	0.267	-0.36	-0.98 – 0.26	0.255			
Birth year [1993]				0.00	-0.01 – 0.01	0.893	-0.01	-0.02 – -0.00	**0.034**	0.70	0.65 – 0.75	**<0.001**	-0.07	-0.20 – 0.06	0.306
Birth year [1994]				0.00	-0.01 – 0.01	0.760	-0.02	-0.03 – -0.01	**<0.001**	0.69	0.64 – 0.74	**<0.001**	0.11	-0.03 – 0.24	0.114
Birth year [1995]				0.00	-0.01 – 0.02	0.382	-0.02	-0.03 – -0.01	**<0.001**	0.69	0.64 – 0.74	**<0.001**	0.11	-0.02 – 0.24	0.110
Birth year [1996]				0.01	0.00 – 0.02	**0.046**	-0.03	-0.04 – -0.02	**<0.001**	0.68	0.63 – 0.73	**<0.001**	0.23	0.11 – 0.36	**<0.001**
Birth year [1997]				0.02	0.01 – 0.03	**0.003**	-0.04	-0.05 – -0.03	**<0.001**	0.67	0.62 – 0.72	**<0.001**	0.14	0.01 – 0.27	**0.030**
Birth year [1998]				0.01	0.00 – 0.02	**0.041**	-0.06	-0.07 – -0.05	**<0.001**	0.65	0.60 – 0.70	**<0.001**	0.13	0.00 – 0.26	**0.049**
Birth year [1999]				0.02	0.01 – 0.03	**0.001**	-0.07	-0.08 – -0.06	**<0.001**	0.64	0.59 – 0.69	**<0.001**	0.11	-0.02 – 0.24	0.085
Birth year [2000]				0.03	0.02 – 0.04	**<0.001**	-0.07	-0.08 – -0.06	**<0.001**	0.64	0.59 – 0.69	**<0.001**	0.25	0.13 – 0.37	**<0.001**

Birth year [2001]	0.04	0.03 – 0.05	**<0.001**	-0.08	-0.09 – -0.07	**<0.001**	0.63	0.58 – 0.68	**<0.001**	0.29	0.17 – 0.42	**<0.001**
Birth year [2002]	0.06	0.05 – 0.07	**<0.001**	-0.07	-0.08 – -0.06	**<0.001**	0.64	0.59 – 0.69	**<0.001**	0.36	0.23 – 0.48	**<0.001**
Mother Income				0.00	0.00 – 0.00	**<0.001**	0.00	0.00 – 0.00	**<0.001**	-0.00	-0.00 – 0.00	0.728
Father Income				0.00	0.00 – 0.00	**<0.001**	0.00	0.00 – 0.00	**<0.001**	-0.00	-0.00 – 0.00	0.336
Mother Education				0.30	0.29 – 0.30	**<0.001**	0.29	0.29 – 0.29	**<0.001**	0.05	0.01 – 0.08	**0.010**
Father Education				0.25	0.25 – 0.26	**<0.001**	0.25	0.25 – 0.26	**<0.001**	0.05	0.02 – 0.09	**0.001**
Father Personality disorder							-0.09	-0.14 – -0.04	**0.001**			
Father Post-traumatic stress disorder							-0.09	-0.13 – -0.05	**<0.001**			
Father Schizophrenia							-0.05	-0.13 – 0.03	0.199			
Father Neuraesthenia							0.00	-0.06 – 0.06	0.982			
Father Affective psychosis							-0.04	-0.08 – 0.01	0.140			
Father Psychological disorder other							-0.09	-0.13 – -0.05	**<0.001**			
Father Hyperkinetic disorder							-0.21	-0.25 – -0.17	**<0.001**			
Father Intellectual disability							-0.28	-0.56 – -0.01	**0.043**			
Father Somatization disorder							-0.03	-0.10 – 0.05	0.465			
Father Anorexia/bulimia							0.10	-0.41 – 0.60	0.711			
Father Suicide/suicide attempt							-0.13	-0.20 – -0.07	**<0.001**			

Father Psychosis NOS	-0.07	-0.15 – 0.01	0.090
Father Organic psychosis other	0.03	-0.10 – 0.16	0.642
Father Dementia	-0.44	-0.71 – -0.18	**0.001**
Mother Personality disorder	-0.13	-0.19 – -0.08	**<0.001**
Mother Post-traumatic stress disorder	-0.08	-0.12 – -0.04	**<0.001**
Mother Schizophrenia	0.03	-0.06 – 0.13	0.506
Mother Neuraesthenia	-0.00	-0.05 – 0.05	0.968
Mother Affective psychosis	-0.01	-0.05 – 0.04	0.686
Mother Psychological disorder other	-0.09	-0.12 – -0.05	**<0.001**
Mother Hyperkinetic disorder	-0.20	-0.25 – -0.16	**<0.001**
Mother Intellectual disability	-0.36	-0.57 – -0.15	**0.001**
Mother Somatization disorder	-0.04	-0.10 – 0.02	0.151
Mother Anorexia/bulimia	-0.05	-0.15 – 0.05	0.363
Mother Suicide/suicide attempt	-0.11	-0.19 – -0.03	**0.010**
Mother Psychosis NOS	-0.04	-0.13 – 0.05	0.370

Mother Organic psychosis other				-0.29	-0.46 – -0.11	**0.001**	
Mother Dementia				-0.08	-0.66 – 0.51	0.800	
Father Drug abuse				-0.08	-0.14 – -0.01	**0.021**	
Father Chronic alcohol abuse				-0.16	-0.25 – -0.08	**<0.001**	
Father Medication abuse				-0.11	-0.23 – -0.00	**0.046**	
Mother Drug abuse				-0.00	-0.07 – 0.07	0.922	
Mother Chronic alcohol abuse				-0.08	-0.18 – 0.03	0.143	
Mother Medication abuse				-0.09	-0.19 – 0.01	0.080	
Observations	667955	667955	667955	607795			5189
R^2 / R^2 adjusted	0.012 / 0.012	0.013 / 0.013	0.185 / 0.185	0.187 / 0.187			0.023 / 0.019

95 % Confidence Interval

Supplemental Table 3

Depression Regression Coefficients

	GPA			GPA			GPA			GPA			GPA		
Predictors	*Estimates*	*CI*	*p*	*Estimates*	*CI*	*p*	*Estimates*	*CI*	*p*	*Estimates*	*CI*	*p*	*Estimates*	*CI*	*p*
(Intercept)	0.04	0.04 – 0.05	**<0.001**	0.03	0.02 – 0.03	**<0.001**	-1.19	-1.20 – -1.19	**<0.001**	-1.89	-1.94 – -1.84	**<0.001**	-0.40	-0.52 – -0.27	**<0.001**
Depressive disorder	-0.41	-0.42 – -0.40	**<0.001**	-0.41	-0.42 – -0.40	**<0.001**	-0.24	-0.25 – -0.23	**<0.001**	-0.20	-0.21 – -0.19	**<0.001**	0.03	-0.11 – 0.17	0.680
Birth order [2]				0.02	0.01 – 0.02	**<0.001**	-0.02	-0.03 – -0.02	**<0.001**	-0.02	-0.03 – -0.02	**<0.001**	-0.06	-0.13 – -0.00	**0.049**
Birth order [3]				-0.00	-0.01 – 0.01	0.758	-0.04	-0.05 – -0.03	**<0.001**	-0.04	-0.05 – -0.03	**<0.001**	-0.40	-0.61 – -0.20	**<0.001**
Birth order [4]				-0.19	-0.22 – -0.16	**<0.001**	-0.11	-0.13 – -0.08	**<0.001**	-0.11	-0.14 – -0.08	**<0.001**	-0.46	-1.40 – 0.47	0.329
Birth order [5]				-0.31	-0.39 – -0.24	**<0.001**	-0.10	-0.17 – -0.04	**0.003**	-0.10	-0.17 – -0.04	**0.002**			
Birth order [6]				-0.28	-0.43 – -0.13	**<0.001**	-0.07	-0.21 – 0.07	0.304	-0.07	-0.21 – 0.06	0.294			
Birth order [7]				-0.14	-0.48 – 0.20	0.412	0.07	-0.24 – 0.38	0.664	0.06	-0.25 – 0.37	0.717			
Birth order [8]				-0.50	-1.18 – 0.18	0.149	-0.35	-0.97 – 0.27	0.271	-0.36	-0.97 – 0.26	0.257			
Birth year [1993]				0.00	-0.01 – 0.01	0.978	-0.01	-0.02 – -0.00	**0.028**	0.70	0.65 – 0.75	**<0.001**	-0.07	-0.20 – 0.06	0.307
Birth year [1994]				0.00	-0.01 – 0.01	0.873	-0.02	-0.03 – -0.01	**<0.001**	0.69	0.64 – 0.74	**<0.001**	0.11	-0.03 – 0.24	0.114
Birth year [1995]				0.00	-0.01 – 0.01	0.509	-0.02	-0.03 – -0.01	**<0.001**	0.69	0.64 – 0.74	**<0.001**	0.11	-0.02 – 0.24	0.110
Birth year [1996]				0.01	-0.00 – 0.02	0.079	-0.03	-0.04 – -0.02	**<0.001**	0.68	0.63 – 0.73	**<0.001**	0.23	0.11 – 0.36	**<0.001**
Birth year [1997]				0.01	0.00 – 0.03	**0.009**	-0.04	-0.05 – -0.03	**<0.001**	0.67	0.62 – 0.72	**<0.001**	0.14	0.01 – 0.27	**0.031**
Birth year [1998]				0.01	-0.00 – 0.02	0.091	-0.06	-0.07 – -0.05	**<0.001**	0.65	0.60 – 0.70	**<0.001**	0.13	0.00 – 0.26	**0.049**
Birth year [1999]				0.02	0.01 – 0.03	**0.002**	-0.07	-0.08 – -0.06	**<0.001**	0.64	0.59 – 0.69	**<0.001**	0.11	-0.02 – 0.24	0.085
Birth year [2000]				0.03	0.02 – 0.04	**<0.001**	-0.07	-0.08 – -0.06	**<0.001**	0.64	0.59 – 0.69	**<0.001**	0.25	0.13 – 0.37	**<0.001**

Birth year [2001]	0.04	0.03 – 0.05	**<0.001**	-0.08	-0.09 – -0.07	**<0.001**	0.63	0.58 – 0.68	**<0.001**	0.29	0.17 – 0.42	**<0.001**
Birth year [2002]	0.06	0.05 – 0.07	**<0.001**	-0.08	-0.09 – -0.06	**<0.001**	0.64	0.59 – 0.69	**<0.001**	0.36	0.23 – 0.48	**<0.001**
Mother Income				0.00	0.00 – 0.00	**<0.001**	0.00	0.00 – 0.00	**<0.001**	-0.00	-0.00 – 0.00	0.728
Father Income				0.00	0.00 – 0.00	**<0.001**	0.00	0.00 – 0.00	**<0.001**	-0.00	-0.00 – 0.00	0.337
Mother Education				0.30	0.29 – 0.30	**<0.001**	0.29	0.29 – 0.29	**<0.001**	0.05	0.01 – 0.08	**0.010**
Father Education				0.26	0.25 – 0.26	**<0.001**	0.25	0.25 – 0.26	**<0.001**	0.05	0.02 – 0.09	**0.001**
Father Personality disorder							-0.11	-0.16 – -0.05	**<0.001**			
Father Post-traumatic stress disorder							-0.10	-0.15 – -0.06	**<0.001**			
Father Schizophrenia							-0.06	-0.14 – 0.01	0.114			
Father Neuraesthenia							-0.01	-0.07 – 0.05	0.761			
Father Affective psychosis							-0.04	-0.09 – 0.00	0.072			
Father Psychological disorder other							-0.10	-0.14 – -0.06	**<0.001**			
Father Hyperkinetic disorder							-0.22	-0.26 – -0.18	**<0.001**			
Father Intellectual disability							-0.29	-0.57 – -0.02	**0.035**			
Father Somatization disorder							-0.05	-0.12 – 0.03	0.214			
Father Anorexia/bulimia							0.10	-0.40 – 0.60	0.698			
Father Suicide/suicide attempt							-0.14	-0.21 – -0.08	**<0.001**			

Father Psychosis NOS	-0.07	-0.15 – 0.01	0.081
Father Organic psychosis other	0.03	-0.10 – 0.16	0.694
Father Dementia	-0.45	-0.72 – -0.18	**0.001**
Mother Personality disorder	-0.14	-0.20 – -0.09	**<0.001**
Mother Post-traumatic stress disorder	-0.09	-0.13 – -0.05	**<0.001**
Mother Schizophrenia	0.02	-0.08 – 0.12	0.697
Mother Neuraesthenia	-0.01	-0.06 – 0.04	0.662
Mother Affective psychosis	-0.02	-0.07 – 0.02	0.311
Mother Psychological disorder other	-0.10	-0.13 – -0.06	**<0.001**
Mother Hyperkinetic disorder	-0.22	-0.26 – -0.18	**<0.001**
Mother Intellectual disability	-0.37	-0.58 – -0.15	**0.001**
Mother Somatization disorder	-0.06	-0.12 – 0.00	0.061
Mother Anorexia/bulimia	-0.06	-0.16 – 0.04	0.251
Mother Suicide/suicide attempt	-0.11	-0.19 – -0.02	**0.011**
Mother Psychosis NOS	-0.05	-0.14 – 0.04	0.299

Mother Organic psychosis other				-0.30	-0.47 – -0.13	**0.001**	
Mother Dementia				-0.16	-0.74 – 0.42	0.594	
Father Drug abuse				-0.09	-0.16 – -0.03	**0.006**	
Father Chronic alcohol abuse				-0.18	-0.26 – -0.09	**<0.001**	
Father Medication abuse				-0.13	-0.24 – -0.02	**0.024**	
Mother Drug abuse				-0.01	-0.08 – 0.06	0.701	
Mother Chronic alcohol abuse				-0.09	-0.19 – 0.02	0.096	
Mother Medication abuse				-0.10	-0.20 – 0.00	0.054	
Observations	667955	667955	667955	607795			5189
R^2 / R^2 adjusted	0.010 / 0.010	0.011 / 0.011	0.185 / 0.185	0.186 / 0.186			0.023 / 0.019

95 % Confidence Interval

Supplemental Table 4

Anxiety Regression Coefficients

	GPA			GPA			GPA			GPA			GPA		
Predictors	*Estimates*	*CI*	*p*	*Estimates*	*CI*	*p*	*Estimates*	*CI*	*p*	*Estimates*	*CI*	*p*	*Estimates*	*CI*	*p*
(Intercept)	0.03	0.03 – 0.03	**<0.001**	0.01	0.00 – 0.02	**0.008**	-1.21	-1.22 – -1.20	**<0.001**	-1.91	-1.96 – -1.86	**<0.001**	-0.39	-0.52 – -0.26	**<0.001**
Anxiety disorder	-0.50	-0.52 – -0.49	**<0.001**	-0.50	-0.52 – -0.48	**<0.001**	-0.26	-0.27 – -0.24	**<0.001**	-0.20	-0.21 – -0.18	**<0.001**	-0.07	-0.36 – 0.21	0.615
Birth order [2]				0.02	0.01 – 0.03	**<0.001**	-0.02	-0.03 – -0.01	**<0.001**	-0.02	-0.03 – -0.02	**<0.001**	-0.06	-0.13 – -0.00	**0.049**
Birth order [3]				-0.00	-0.01 – 0.01	0.744	-0.04	-0.05 – -0.03	**<0.001**	-0.04	-0.05 – -0.03	**<0.001**	-0.41	-0.61 – -0.20	**<0.001**
Birth order [4]				-0.19	-0.22 – -0.16	**<0.001**	-0.11	-0.14 – -0.08	**<0.001**	-0.11	-0.14 – -0.08	**<0.001**	-0.47	-1.40 – 0.47	0.327
Birth order [5]				-0.32	-0.39 – -0.25	**<0.001**	-0.11	-0.17 – -0.04	**0.002**	-0.10	-0.17 – -0.04	**0.002**			
Birth order [6]				-0.29	-0.44 – -0.14	**<0.001**	-0.08	-0.22 – 0.06	0.252	-0.08	-0.21 – 0.06	0.260			
Birth order [7]				-0.19	-0.53 – 0.15	0.267	0.04	-0.27 – 0.35	0.793	0.04	-0.27 – 0.34	0.820			
Birth order [8]				-0.54	-1.22 – 0.14	0.121	-0.37	-0.99 – 0.25	0.245	-0.38	-0.99 – 0.24	0.234			
Birth Year [1993]				0.00	-0.01 – 0.01	0.946	-0.01	-0.02 – -0.00	**0.029**	0.70	0.65 – 0.75	**<0.001**	-0.07	-0.20 – 0.06	0.306
Birth Year [1994]				0.00	-0.01 – 0.01	0.816	-0.02	-0.03 – -0.01	**<0.001**	0.70	0.65 – 0.75	**<0.001**	0.11	-0.03 – 0.24	0.111
Birth Year [1995]				0.00	-0.01 – 0.01	0.545	-0.02	-0.03 – -0.01	**<0.001**	0.69	0.64 – 0.74	**<0.001**	0.11	-0.02 – 0.24	0.107
Birth Year [1996]				0.01	-0.00 – 0.02	0.092	-0.03	-0.04 – -0.02	**<0.001**	0.68	0.63 – 0.73	**<0.001**	0.23	0.11 – 0.36	**<0.001**
Birth Year [1997]				0.01	0.00 – 0.03	**0.011**	-0.04	-0.05 – -0.03	**<0.001**	0.68	0.63 – 0.73	**<0.001**	0.14	0.01 – 0.27	**0.030**
Birth Year [1998]				0.01	-0.00 – 0.02	0.072	-0.06	-0.07 – -0.05	**<0.001**	0.66	0.61 – 0.71	**<0.001**	0.13	0.00 – 0.26	**0.049**
Birth Year [1999]				0.02	0.01 – 0.03	**0.002**	-0.07	-0.08 – -0.06	**<0.001**	0.65	0.60 – 0.70	**<0.001**	0.11	-0.02 – 0.24	0.084
Birth Year [2000]				0.03	0.02 – 0.04	**<0.001**	-0.07	-0.08 – -0.06	**<0.001**	0.64	0.59 – 0.69	**<0.001**	0.25	0.13 – 0.37	**<0.001**

Birth Year [2001]	0.04	0.03 – 0.05	**<0.001**	-0.08	-0.09 – -0.07	**<0.001**	0.64	0.59 – 0.69	**<0.001**	0.29	0.17 – 0.42	**<0.001**
Birth Year [2002]	0.06	0.05 – 0.07	**<0.001**	-0.08	-0.09 – -0.07	**<0.001**	0.64	0.59 – 0.69	**<0.001**	0.36	0.23 – 0.48	**<0.001**
Mother Income				0.00	0.00 – 0.00	**<0.001**	0.00	0.00 – 0.00	**<0.001**	-0.00	-0.00 – 0.00	0.713
Father Income				0.00	0.00 – 0.00	**<0.001**	0.00	0.00 – 0.00	**<0.001**	-0.00	-0.00 – 0.00	0.322
Mother Education				0.30	0.29 – 0.30	**<0.001**	0.29	0.29 – 0.30	**<0.001**	0.05	0.01 – 0.08	**0.010**
Father Education				0.26	0.25 – 0.26	**<0.001**	0.25	0.25 – 0.26	**<0.001**	0.05	0.02 – 0.09	**0.002**
Father Personality disorder							-0.13	-0.19 – -0.08	**<0.001**			
Father Post-traumatic stress disorder							-0.15	-0.19 – -0.11	**<0.001**			
Father Schizophrenia							-0.07	-0.14 – 0.01	0.091			
Father Neuraesthenia							-0.04	-0.11 – 0.02	0.162			
Father Affective psychosis							-0.10	-0.14 – -0.05	**<0.001**			
Father Psychological disorder other							-0.13	-0.17 – -0.09	**<0.001**			
Father Hyperkinetic disorder							-0.25	-0.29 – -0.21	**<0.001**			
Father Intellectual disability							-0.29	-0.56 – -0.02	**0.038**			
Father Somatization disorder							-0.08	-0.15 – -0.00	**0.039**			
Father Anorexia/bulimia							0.03	-0.48 – 0.53	0.911			
Father Suicide/suicide attempt							-0.18	-0.25 – -0.12	**<0.001**			

Father Psychosis NOS	-0.08	-0.16 – -0.00	**0.047**
Father Organic psychosis other	-0.01	-0.14 – 0.12	0.867
Father Dementia	-0.50	-0.76 – -0.23	**<0.001**
Mother Personality disorder	-0.17	-0.23 – -0.11	**<0.001**
Mother Post-traumatic stress disorder	-0.13	-0.17 – -0.09	**<0.001**
Mother Schizophrenia	0.03	-0.07 – 0.13	0.535
Mother Neuraesthenia	-0.06	-0.10 – -0.01	**0.022**
Mother Affective psychosis	-0.06	-0.10 – -0.01	**0.012**
Mother Psychological disorder other	-0.13	-0.17 – -0.09	**<0.001**
Mother Hyperkinetic disorder	-0.25	-0.29 – -0.20	**<0.001**
Mother Intellectual disability	-0.36	-0.57 – -0.14	**0.001**
Mother Somatization disorder	-0.09	-0.15 – -0.03	**0.003**
Mother Anorexia/bulimia	-0.10	-0.20 – -0.00	**0.049**
Mother Suicide/suicide attempt	-0.15	-0.23 – -0.06	**<0.001**
Mother Psychosis NOS	-0.05	-0.13 – 0.04	0.311

Mother Organic psychosis other				-0.31	-0.49 – -0.14	**<0.001**	
Mother Dementia				-0.11	-0.69 – 0.47	0.715	
Father Drug abuse				-0.09	-0.16 – -0.02	**0.009**	
Father Chronic alcohol abuse				-0.19	-0.27 – -0.10	**<0.001**	
Father Medication abuse				-0.11	-0.23 – 0.00	0.050	
Mother Drug abuse				0.01	-0.06 – 0.08	0.776	
Mother Chronic alcohol abuse				-0.08	-0.18 – 0.02	0.119	
Mother Medication abuse				-0.09	-0.19 – 0.01	0.079	
Observations	667955	667955	667955	607795			5189
R^2 / R^2 adjusted	0.005 / 0.005	0.006 / 0.006	0.183 / 0.183	0.185 / 0.185			0.023 / 0.019

95 % Confidence Interval

Supplemental Table 5

Mother Internalizing Regression Coefficients

	GPA			GPA			GPA			GPA			GPA		
Predictors	*Estimates*	*CI*	*p*	*Estimates*	*CI*	*p*	*Estimates*	*CI*	*p*	*Estimates*	*CI*	*p*	*Estimates*	*CI*	*p*
(Intercept)	0.05	0.04 – 0.05	**<0.001**	0.02	0.02 – 0.03	**<0.001**	-1.19	-1.20 – -1.18	**<0.001**	-1.87	-1.92 – -1.82	**<0.001**	-0.39	-0.52 – -0.26	**<0.001**
Mother Internalizing	-0.45	-0.46 – -0.43	**<0.001**	-0.44	-0.46 – -0.43	**<0.001**	-0.25	-0.26 – -0.24	**<0.001**	-0.21	-0.22 – -0.20	**<0.001**	-0.06	-0.22 – 0.10	0.467
Birth order [2]				0.02	0.01 – 0.02	**<0.001**	-0.02	-0.03 – -0.02	**<0.001**	-0.02	-0.03 – -0.02	**<0.001**	-0.06	-0.13 – -0.00	**0.049**
Birth order [3]				-0.00	-0.02 – 0.01	0.426	-0.04	-0.05 – -0.03	**<0.001**	-0.05	-0.06 – -0.04	**<0.001**	-0.41	-0.61 – -0.20	**<0.001**
Birth order [4]				-0.19	-0.22 – -0.16	**<0.001**	-0.11	-0.14 – -0.08	**<0.001**	-0.11	-0.14 – -0.08	**<0.001**	-0.47	-1.40 – 0.46	0.326
Birth order [5]				-0.32	-0.40 – -0.25	**<0.001**	-0.11	-0.18 – -0.04	**0.001**	-0.11	-0.18 – -0.04	**0.001**			
Birth order [6]				-0.29	-0.44 – -0.14	**<0.001**	-0.08	-0.21 – 0.06	0.275	-0.08	-0.22 – 0.06	0.268			
Birth order [7]				-0.15	-0.49 – 0.20	0.401	0.08	-0.23 – 0.39	0.620	0.07	-0.24 – 0.38	0.655			
Birth order [8]				-0.50	-1.18 – 0.18	0.151	-0.35	-0.96 – 0.27	0.272	-0.36	-0.97 – 0.26	0.256			
Birth year [1993]				0.00	-0.01 – 0.01	0.949	-0.01	-0.02 – -0.00	**0.030**	0.68	0.63 – 0.73	**<0.001**	-0.07	-0.20 – 0.06	0.301
Birth year [1994]				0.00	-0.01 – 0.01	0.682	-0.02	-0.03 – -0.01	**<0.001**	0.67	0.62 – 0.73	**<0.001**	0.11	-0.03 – 0.24	0.111
Birth year [1995]				0.01	-0.01 – 0.02	0.335	-0.02	-0.03 – -0.01	**<0.001**	0.67	0.62 – 0.72	**<0.001**	0.11	-0.02 – 0.24	0.109
Birth year [1996]				0.01	0.00 – 0.02	**0.045**	-0.03	-0.04 – -0.02	**<0.001**	0.66	0.61 – 0.71	**<0.001**	0.23	0.11 – 0.36	**<0.001**
Birth year [1997]				0.02	0.01 – 0.03	**0.003**	-0.04	-0.05 – -0.03	**<0.001**	0.66	0.61 – 0.71	**<0.001**	0.14	0.01 – 0.27	**0.030**
Birth year [1998]				0.01	0.00 – 0.02	**0.028**	-0.06	-0.07 – -0.05	**<0.001**	0.64	0.58 – 0.69	**<0.001**	0.13	0.00 – 0.26	**0.049**
Birth year [1999]				0.02	0.01 – 0.03	**0.001**	-0.07	-0.08 – -0.06	**<0.001**	0.62	0.57 – 0.68	**<0.001**	0.11	-0.02 – 0.24	0.084

Birth year [2000]	0.03	0.02 – 0.04	**<0.001**	-0.07	-0.08 – -0.06	**<0.001**	0.62	0.57 – 0.67	**<0.001**	0.25	0.13 – 0.37	**<0.001**
Birth year [2001]	0.04	0.03 – 0.05	**<0.001**	-0.08	-0.09 – -0.07	**<0.001**	0.61	0.56 – 0.67	**<0.001**	0.29	0.17 – 0.42	**<0.001**
Birth year [2002]	0.06	0.05 – 0.07	**<0.001**	-0.07	-0.08 – -0.06	**<0.001**	0.62	0.57 – 0.67	**<0.001**	0.36	0.23 – 0.48	**<0.001**
Mother Income				0.00	0.00 – 0.00	**<0.001**	0.00	0.00 – 0.00	**<0.001**	-0.00	-0.00 – 0.00	0.703
Father Income				0.00	0.00 – 0.00	**<0.001**	0.00	0.00 – 0.00	**<0.001**	-0.00	-0.00 – 0.00	0.314
Mother Education				0.30	0.29 – 0.30	**<0.001**	0.29	0.29 – 0.29	**<0.001**	0.05	0.01 – 0.09	**0.010**
Father Education				0.26	0.25 – 0.26	**<0.001**	0.25	0.25 – 0.26	**<0.001**	0.05	0.02 – 0.09	**0.002**
Father Personality disorder							-0.11	-0.16 – -0.06	**<0.001**			
Father Post-traumatic stress disorder							-0.10	-0.15 – -0.06	**<0.001**			
Father Schizophrenia							-0.05	-0.13 – 0.02	0.171			
Father Neuraesthenia							-0.01	-0.08 – 0.05	0.674			
Father Affective psychosis							-0.05	-0.10 – -0.00	**0.032**			
Father Psychological disorder other							-0.11	-0.14 – -0.07	**<0.001**			
Father Hyperkinetic disorder							-0.23	-0.27 – -0.18	**<0.001**			
Father Intellectual disability							-0.30	-0.57 – -0.03	**0.032**			
Father Somatization disorder							-0.04	-0.12 – 0.03	0.247			

Father Anoreksia/bulimia	0.06	-0.44 – 0.57	0.807
Father Suicide/suicide attempt	-0.16	-0.23 – -0.10	**<0.001**
Father Psychosis nos	-0.06	-0.14 – 0.02	0.126
Father Organic psychosis other	0.01	-0.12 – 0.14	0.922
Father Dementia	-0.45	-0.72 – -0.18	**0.001**
Mother Personality disorder	-0.14	-0.19 – -0.08	**<0.001**
Mother Post-traumatic stress disorder	-0.10	-0.13 – -0.06	**<0.001**
Mother Schizophrenia	0.01	-0.09 – 0.10	0.883
Mother Neuraesthenia	-0.04	-0.08 – 0.01	0.141
Mother Affective psychosis	-0.04	-0.08 – 0.01	0.119
Mother Psychological disorder other	-0.10	-0.14 – -0.06	**<0.001**
Mother Hyperkinetic disorder	-0.23	-0.27 – -0.18	**<0.001**
Mother Intellectual disability	-0.35	-0.56 – -0.13	**0.001**
Mother Somatization disorder	-0.06	-0.12 – -0.00	**0.042**

Mother Anoreksia/bulimia										-0.06	-0.16 – 0.04	0.223			
Mother Suicide/suicide attempt										-0.11	-0.19 – -0.03	**0.010**			
Mother Psychosis nos										-0.05	-0.14 – 0.04	0.251			
Mother Organic psychosis other										-0.30	-0.47 – -0.13	**0.001**			
Mother Dementia										-0.13	-0.71 – 0.45	0.663			
Father Drug abuse										-0.09	-0.16 – -0.02	**0.008**			
Father Chronic alcohol abuse										-0.17	-0.26 – -0.08	**<0.001**			
Father Medication abuse										-0.12	-0.24 – -0.01	**0.032**			
Mother Drug abuse										-0.00	-0.07 – 0.07	0.902			
Mother Chronic alcohol abuse										-0.08	-0.18 – 0.03	0.146			
Mother Medication abuse										-0.09	-0.18 – 0.01	0.089			
Observations	665881			665881			665881			605811			5189		
R^2 / R^2 adjusted	0.010 / 0.010			0.010 / 0.010			0.185 / 0.185			0.187 / 0.187			0.023 / 0.019		

95 % Confidence Interval

Supplemental Table 6

Father Internalizing Regression Coefficients

	GPA			GPA			GPA			GPA			GPA		
Predictors	*Estimates*	*CI*	*p*	*Estimates*	*CI*	*p*	*Estimates*	*CI*	*p*	*Estimates*	*CI*	*p*	*Estimates*	*CI*	*p*
(Intercept)	0.05	0.05 – 0.06	**<0.001**	0.03	0.02 – 0.04	**<0.001**	-1.18	-1.19 – -1.17	**<0.001**	-1.83	-1.88 – -1.77	**<0.001**	-0.39	-0.52 – -0.26	**<0.001**
Father Internalizing	-0.41	-0.42 – -0.40	**<0.001**	-0.41	-0.42 – -0.40	**<0.001**	-0.23	-0.24 – -0.22	**<0.001**	-0.18	-0.20 – -0.17	**<0.001**	-0.04	-0.21 – 0.13	0.635
Birth order [2]				0.01	0.00 – 0.01	**0.021**	-0.03	-0.03 – -0.02	**<0.001**	-0.03	-0.04 – -0.03	**<0.001**	-0.06	-0.13 – -0.00	**0.049**
Birth order [3]				-0.01	-0.02 – -0.00	**0.023**	-0.05	-0.06 – -0.04	**<0.001**	-0.05	-0.06 – -0.04	**<0.001**	-0.41	-0.61 – -0.20	**<0.001**
Birth order [4]				-0.19	-0.22 – -0.16	**<0.001**	-0.11	-0.14 – -0.09	**<0.001**	-0.11	-0.14 – -0.09	**<0.001**	-0.47	-1.40 – 0.47	0.326
Birth order [5]				-0.31	-0.38 – -0.24	**<0.001**	-0.10	-0.17 – -0.03	**0.004**	-0.10	-0.17 – -0.03	**0.004**			
Birth order [6]				-0.30	-0.46 – -0.15	**<0.001**	-0.10	-0.24 – 0.04	0.162	-0.10	-0.24 – 0.04	0.155			
Birth order [7]				-0.18	-0.52 – 0.16	0.292	0.04	-0.27 – 0.35	0.789	0.03	-0.27 – 0.34	0.833			
Birth order [8]				-0.57	-1.24 – 0.11	0.102	-0.39	-1.00 – 0.23	0.217	-0.39	-1.01 – 0.22	0.208			
Birth year [1993]				0.00	-0.01 – 0.01	0.740	-0.01	-0.02 – 0.00	0.057	0.65	0.59 – 0.70	**<0.001**	-0.07	-0.20 – 0.06	0.303
Birth year [1994]				0.01	-0.01 – 0.02	0.336	-0.02	-0.03 – -0.01	**0.002**	0.64	0.59 – 0.70	**<0.001**	0.11	-0.03 – 0.24	0.112
Birth year [1995]				0.01	0.00 – 0.02	**0.041**	-0.02	-0.03 – -0.01	**0.001**	0.64	0.59 – 0.70	**<0.001**	0.11	-0.02 – 0.24	0.109
Birth year [1996]				0.02	0.01 – 0.03	**<0.001**	-0.02	-0.03 – -0.01	**<0.001**	0.64	0.58 – 0.69	**<0.001**	0.23	0.11 – 0.36	**<0.001**
Birth year [1997]				0.03	0.01 – 0.04	**<0.001**	-0.03	-0.04 – -0.02	**<0.001**	0.63	0.57 – 0.69	**<0.001**	0.14	0.01 – 0.27	**0.030**
Birth year [1998]				0.02	0.01 – 0.03	**<0.001**	-0.05	-0.06 – -0.04	**<0.001**	0.61	0.55 – 0.66	**<0.001**	0.13	0.00 – 0.26	**0.050**

Birth year [1999]	0.03	0.02 – 0.04	**<0.001**	-0.06	-0.07 – -0.05	**<0.001**	0.60	0.54 – 0.65	**<0.001**	0.11	-0.02 – 0.24	0.085
Birth year [2000]	0.04	0.03 – 0.05	**<0.001**	-0.07	-0.08 – -0.06	**<0.001**	0.60	0.54 – 0.65	**<0.001**	0.25	0.13 – 0.37	**<0.001**
Birth year [2001]	0.05	0.04 – 0.06	**<0.001**	-0.08	-0.09 – -0.07	**<0.001**	0.59	0.53 – 0.64	**<0.001**	0.29	0.17 – 0.42	**<0.001**
Birth year [2002]	0.07	0.06 – 0.08	**<0.001**	-0.07	-0.08 – -0.06	**<0.001**	0.59	0.54 – 0.65	**<0.001**	0.36	0.23 – 0.48	**<0.001**
Mother Income				0.00	0.00 – 0.00	**<0.001**	0.00	0.00 – 0.00	**<0.001**	-0.00	-0.00 – 0.00	0.714
Father Income				0.00	0.00 – 0.00	**<0.001**	0.00	0.00 – 0.00	**<0.001**	-0.00	-0.00 – 0.00	0.318
Mother Education				0.30	0.29 – 0.30	**<0.001**	0.29	0.29 – 0.29	**<0.001**	0.05	0.01 – 0.08	**0.010**
Father Education				0.26	0.25 – 0.26	**<0.001**	0.25	0.25 – 0.26	**<0.001**	0.05	0.02 – 0.09	**0.002**
Father Personality disorder							-0.13	-0.18 – -0.08	**<0.001**			
Father Post-traumatic stress disorder							-0.15	-0.19 – -0.10	**<0.001**			
Father Schizophrenia							-0.09	-0.17 – -0.02	**0.018**			
Father Neuraesthenia							-0.05	-0.12 – 0.01	0.091			
Father Affective psychosis							-0.08	-0.13 – -0.04	**0.001**			
Father Psychological disorder other							-0.13	-0.17 – -0.10	**<0.001**			
Father Hyperkinetic disorder							-0.26	-0.30 – -0.22	**<0.001**			
Father Intellectual disability							-0.33	-0.60 – -0.05	**0.019**			

Father Somatization disorder	-0.07	-0.15 – 0.00	0.052
Father Anoreksia/bulimia	0.05	-0.45 – 0.55	0.857
Father Suicide/suicide attempt	-0.16	-0.23 – -0.10	**<0.001**
Father Psychosis nos	-0.08	-0.16 – -0.00	**0.040**
Father Organic psychosis other	-0.00	-0.13 – 0.13	0.987
Father Dementia	-0.51	-0.77 – -0.25	**<0.001**
Mother Personality disorder	-0.19	-0.25 – -0.13	**<0.001**
Mother Post-traumatic stress disorder	-0.09	-0.13 – -0.05	**<0.001**
Mother Schizophrenia	0.04	-0.06 – 0.14	0.411
Mother Neuraesthenia	-0.03	-0.08 – 0.02	0.253
Mother Affective psychosis	-0.04	-0.09 – 0.00	0.061
Mother Psychological disorder other	-0.10	-0.14 – -0.06	**<0.001**
Mother Hyperkinetic disorder	-0.24	-0.28 – -0.19	**<0.001**
Mother Intellectual disability	-0.35	-0.59 – -0.12	**0.003**

Mother Somatization disorder										-0.05	-0.11 – 0.02	0.134			
Mother Anoreksia/bulimia										-0.11	-0.21 – -0.00	**0.047**			
Mother Suicide/suicide attempt										-0.14	-0.23 – -0.06	**0.001**			
Mother Psychosis nos										-0.00	-0.10 – 0.09	0.934			
Mother Organic psychosis other										-0.32	-0.50 – -0.14	**0.001**			
Mother Dementia										-0.12	-0.70 – 0.46	0.689			
Father Drug abuse										-0.12	-0.18 – -0.05	**0.001**			
Father Chronic alcohol abuse										-0.19	-0.28 – -0.11	**<0.001**			
Father Medication abuse										-0.12	-0.24 – -0.01	**0.030**			
Mother Drug abuse										0.01	-0.06 – 0.09	0.705			
Mother Chronic alcohol abuse										-0.10	-0.21 – 0.01	0.063			
Mother Medication abuse										-0.12	-0.22 – -0.02	**0.022**			
Observations	649315			649315			649315			590321			5189		
R^2 / R^2 adjusted	0.007 / 0.007			0.008 / 0.008			0.185 / 0.185			0.187 / 0.187			0.023 / 0.019		

95 % Confidence Interval

Supplemental Table 7

Sibling Analysis Regression Coefficients

		**GPA**	
Predictors	*Estimates*	*CI*	*p*
Internalizing 11–13	-0.00	-0.02 – 0.02	0.968
Internalizing 14–16	-0.03	-0.05 – -0.01	**<0.001**
Birth order [2]	-0.16	-0.19 – -0.14	**<0.001**
Birth order [3]	-0.23	-0.27 – -0.19	**<0.001**
Birth order [4]	-0.28	-0.35 – -0.21	**<0.001**
Birth order [5]	-0.27	-0.42 – -0.13	**<0.001**
Birth order [6]	-0.27	-0.54 – -0.00	**0.048**
Birth order [7]	0.14	-0.45 – 0.73	0.632
Birth order [8]	-0.41	-1.31 – 0.48	0.362
Birth year [1993]	0.25	0.12 – 0.38	**<0.001**
Birth year [1994]	0.28	0.16 – 0.41	**<0.001**
Birth year [1995]	0.31	0.19 – 0.44	**<0.001**
Birth year [1996]	0.33	0.20 – 0.46	**<0.001**

Birth year [1997]	0.35	0.22 – 0.48	**<0.001**
Birth year [1998]	0.37	0.24 – 0.50	**<0.001**
Birth year [1999]	0.39	0.26 – 0.52	**<0.001**
Birth year [2000]	0.41	0.27 – 0.55	**<0.001**
Birth year [2001]	0.45	0.31 – 0.59	**<0.001**
Birth year [2002]	0.46	0.32 – 0.60	**<0.001**
Observations	73740		
R^2 / R^2 adjusted	0.010 / -0.779		

95 % Confidence Interval

Supplemental Table 8

Mean GPA (Grade Point Average)

Sample	No Internalizing	Internalizing	Difference
Population	0.05	-0.38	0.43
Adoptee	-0.01	0.01	-0.03
Sibling Comparison	-0.23	-0.27	0.04

Supplemental Table 9

Mean GPA (Grade Point Average) by Gender

Gender	Sample	No Internalizing	Internalizing	Difference
Boys	Population	-0.19	-0.60	0.41
Girls	Population	0.30	-0.15	0.44
Boys	Adoptee	-0.37	-0.37	-0.01
Girls	Adoptee	0.26	0.27	-0.01
Boys	Sibling Comparison	-0.44	-0.49	0.04
Girls	Sibling Comparison	-0.02	-0.04	0.02
